# Research on Grading Detection of the Wheat Seeds

**DOI:** 10.1155/2014/702906

**Published:** 2014-04-16

**Authors:** Xian-Zhong Han, Ke-Jian Wang, Yingchun Yuan, Chen Chen, Liang Liang

**Affiliations:** School of Information Science and Technology, Agricultural University of Hebei, Baoding 071002, China

## Abstract

Evaluating the merits of the wheat seed is an important significance for wheat breeding. We studied analytic hierarchy process (AHP) for seeds grading by digital image processing techniques in the paper. Firstly, preprocess the collected wheat seed images; extract some parameters, such as area, plumpness, rectangular, and elongation of the seed, and then build the level model. Experiments showed the model is right, and level accuracy rate is more than 95%.

## 1. Introduction

Wheat is one of the most important grains in the world, which yield ranks 2 after rice. It is a major food resource and an important industrial raw material as well. The good and bad wheat gain is not only the determining factor for yield and quality but is also the comprehensive index of species adaptations. Identifying and grading of wheat seed size and plumpness have important significance for improving wheat quality and promoting the development of the wheat industrial chain.

There is a lot of research in wheat with image processing. In 1985, Zayas et al. [1, 2] distinguished between two varieties of winter wheat by visual images. In 1987, Sapirstein [3] studied the classification of cereal crops and designed classifier. In 1989, Neuman et al. [4] analyzed the color feature of wheat seed by image processing. In 2000, Jayas et al. [5, 6] classified wheat by the texture characteristics and established the texture feature model. In 2005, Ferrio et al. [7] forecasted wheat yield according to visible and near-infrared reflectance spectra. Domestically, in 2004, Guiqin et al. [8], from China Agricultural University, extracted wheat image morphological and color characteristic parameters to establish a yield dynamic model and forecasted wheat yield. In 2005, HongWei [9] judged the level quality of wheat by color, shape, and texture based on signal processing method. In 2011, Kun et al. [10] extracted some characteristics of the wheat awn number, awn length, and wheat spike length color and identified four spring wheat varieties from Xinjiang by BP neural network. Xu et al. [11, 12] classified four wheat varieties by median filtering and threshold method.

On the whole, it is wide about wheat researches based on image processing, but most of them are limited to species identification, and it is rare on wheat seed grading. Reference [4] mentions a method with the signal processing to determine wheat quality, which requires amount of data and long time as well as expensive equipment. Aiming at the existing problems in the detection of agricultural products, we put forward the level method of wheat seed quality based on AHP and lay the foundation for the application of wheat grading detection technology.

## 2. Image Capturing and Preprocessing 

### 2.1. Image Capturing

Images are taken by Sony DSC-WX50 digital camera, which is equipped with 16 million pixels and the 3x optical zoom camera. The actual images are about 9 million pixels and saved as BMP format.

When images are captured, we select plain black wood as background, adequate illumination, and then make wheat seed drying tile based on the background. In order to prevent the shadow on image segmentation, incandescent light is filled with illumination in the shadow side. The camera, fixed on the solid support, vertically shoots wheat seed with 3x optical macrozoom mode and shoots the wheat seed of different densities at the same conditions.

### 2.2. Preprocessing of Wheat Seeds Images

In order to get wheat seed features accurately, wheat seed images are preprocessed through grayscale, enhancement, smoothing (denoising), binarization, and segmentation; finally, we obtain the desired images.

**Figure 1:**
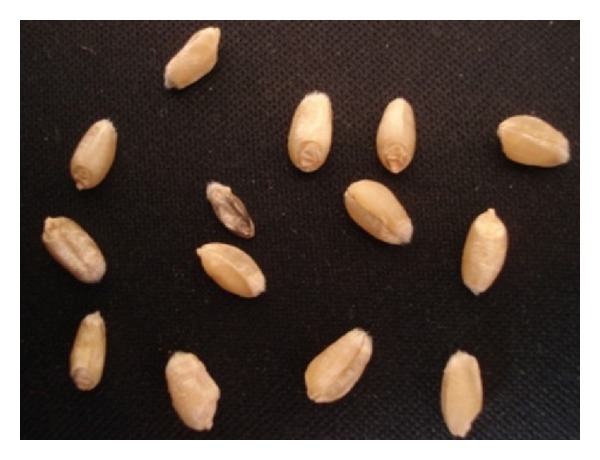
The original image of wheat seeds.

**Figure 2:**
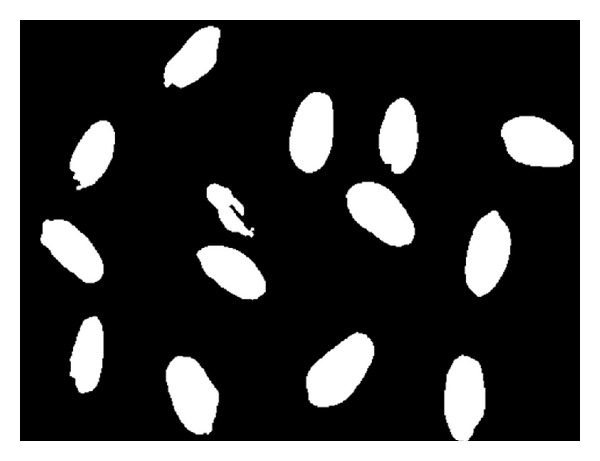
Preprocessing results.

The images are processed by gray scale to simplify the original image color, so that the seed seeds are separated more easily from the background. The images are enhanced by the Gauss 5 × 5 filter template, which can effectively eliminate the noise and preserve the image detail. Images are segmented by Otsu method to optimize threshold; histogram and threshold make the wheat binarization images separated from the background, which lays the foundation for the gain feature extraction.  Figure 1 is the original wheat gain image; Figure 2 is the one after preprocessing.

## 3. Feature Extractions of Wheat Seeds 

Feature extraction is a key step in wheat seed grading; the goodness and badness of wheat seed are mainly reflected by geometric features. In this paper we judge the wheat seed level by extracting geometric features of wheat seed, including perimeter, area, and elongation.

### 3.1. Perimeter Extraction

Perimeter is a measure of the size of an object appearance, under normal circumstances. In general, the better the quality of wheat is, the larger the perimeter is. Firstly, the paper extracts and tracks the image edge and gets seed profile; and then it chooses 8-connectivity chain code method to calculate the perimeter. 8-connectivity chain code method is exactly in line with the actual situation of pixels that can accurately describe the information center of the pixel and its neighbors. The formula based on 8-connectivity chain code is as follows:

(1) $$\begin{array}{c} P=\text{sqrt}\left(2\right)\times N_{d}+N_{x}+N_{y}. \end{array}$$

In this formula, *N*_*d*_ represents the number of chain code in the diagonal direction; *N*_*x*_ represents the number of pixels in the horizontal direction; *N*_*y*_ represents the number of pixels in the vertical direction; *N*_*x*_ + *N*_*y*_ represents the number of even chain code.

### 3.2. Area Extraction

Area is a measure of the size of plane taken by an object, and it is a metric evaluation for object size in the two-dimensional space. A high-quality seed looks smooth and plump; when it is projected onto a two-dimensional plane, the area will be larger.

Wheat area extraction is simple, which calculates the number of pixels taken by wheat seed directly; the length of each pixel is 1.

### 3.3. Circularity Extraction

Circularity indicates deviation degree between an object and a standard circle, which is determined by the perimeter and area of the object and represented by *C*. Wheat seed circularity is similar with plumpness. High-quality wheat is ripe and plump with cylindrical appearance. The larger the deviation degree between an irregular object and a standard circle, the lesser the circularity. Circularity is defined by the following equation:

(2) $$\begin{array}{c} C=\frac{4\pi A}{P^{2}}. \end{array}$$

In this formula, *A* represents the area of wheat seed; *P* represents the perimeter of wheat seeds.

### 3.4. Elongation Extraction

Elongation, also called eccentricity, is the ratio of the minor axis and the major axis of the target image, which describes stretch ability and slenderness of the image. Elongation is usually used for the slender objects apart from circular and square object and reflects partly the compact degree of the target object. Its symbol is *E*, which is calculated as

(3) $$\begin{array}{c} E=\frac{H}{W}. \end{array}$$

In this formula, *H* represents the major axis of wheat seeds; *W* represents the minor axis of wheat seeds.

### 3.5. Rectangularity Extraction

Rectangularity indicates the external smallest rectangle of target object and reflects the filling degree. The object placement angle has great influence on external rectangle. Its symbol is *R*, which is calculated as

(4) $$\begin{array}{c} R=\frac{A}{\left(H\times W\right)}. \end{array}$$

In this formula, *A* represents the area of wheat seed; *H* represents the major axis of wheat seed; *W* represents the minor axis of wheat seed.

## 4. The Model of the Wheat Seeds Grading

### 4.1. The Analytic Hierarchy Process

The analytic hierarchy process, called AHP for short, is a combination of qualitative, quantitative, systematic, and hierarchical analysis methods.

AHP is a complex problem which is decomposed into a number of layers and compares the effect between various layers or factors. According to the influence, AHP establishes judgment matrix, calculates maximum feature value and feature vector matrix, obtains the weight coefficients of importance from different solutions, and compares the weight coefficients to get the best solution.

### 4.2. Steps of AHP Model

#### 4.2.1. Building Comparison Matrix

Assuming there are *n* factors in a layer, *X* = {*x*_1_, *x*_2_,…, *x*_*n*_} is to compare the extent of their impact on a criterion (or target) of upper layer and determine the proportion of the layer relative to the criteria (i.e., sort the influence degrees of the *N* factors on the target of the upper layer). *a*_*ij*_ represents the comparison on result of the number *i* factor relative to the number *j* factor

(5) $$\begin{array}{c} A={\left(a_{ij}\right)}_{n\times n}=\left(\begin{array}{cccc} a_{11} & a_{12} & \cdots & a_{1n}\\ a_{21} & a_{22} & \cdots & a_{2n}\\ \vdots & \vdots & \vdots & \vdots \\ a_{n1} & a_{n2} & \cdots & a_{nn} \end{array}\right). \end{array}$$

In this formula, *A* is called paired comparison matrix, *a*_*ij*_ = 1/*a*_*ji*_.

#### 4.2.2. Hierarchy Single Ranking and Its Consistency Test

Hierarchy single ranking is the process of determining degree that the factors of underlying factors influence a factor of the upper layer, with weight value to indicate the degree. For each pairwise comparison matrix, it calculates the maximum eigen value and the eigen vector and then makes the consistency test with consistency index, random consistency index, and consistency ratio. If it passes the tests, normalized eigen vector is the weight value; otherwise, it has to rebuild the paired comparison matrix.

#### 4.2.3. Hierarchy General Ranking and Its Consistency Test

Hierarchy general ranking is the sorting process of determining degree that the factors of one factor influence the overall goal; the sorting process is from the top to the lowest layer by layer. It calculates the weight vector of sorting results from the top to the lowest and uses consistency ratio of hierarchy general ranking for testing. If the test is passed, it can make decisions by weight vector of general ranking; otherwise it has to rethink the model to reconstruct the paired comparison matrices of greater consistency ratios.

### 4.3. AHP Model of Wheat Seed Grading

#### 4.3.1. Extraction Characteristic Parameters of Wheat

To facilitate the presentation, we select 14 wheat seeds with the numbers 1–14, and then they are divided into three different levels: excellent, good, and poor. There are 6 excellent seeds, 3 good seeds, and 4 poor seeds. Through pretreatment of wheat seed and the image feature extraction, the image characteristic values of wheat seeds are obtained, which are shown in Table 1.

In Table 1, Num is seeds number, *A* is area, *P* is perimeter, *C* is circularity, *E* is elongation, and *R* is rectangularity.

Considering area, perimeter, circularity, elongation, and rectangularity, a comprehensive evaluation model is constructed at last. Target layer is to determine the wheat seed; criterion layer is the factors including area, perimeter, circularity, elongation, and rectangularity; project layer is the wheat seeds.

#### 4.3.2. Building Paired Comparison Matrix

According to AHP, it is easy to build judgment matrix. The method of constructing judgment matrix is as follows: every downward subordinate element (called criteria) is considered as the first element of judgment matrix (located in the upper left corner of the matrix); elements attached to it are orderly arranged in the first row and the first column followed.

The method of filling in the matrix is as follows: according to the matrix criteria, compare all of the elements with each other and calculate degree of importance between two elements; then assign the degree of importance by 1–9 (importance scale in Table 2).

The importance analysis of the judgment matrix is as follows: the extractions of area, perimeter, and elongation are independent; the extraction of circularity is not independent and based on area and perimeter; the extraction of rectangularity is not independent too, which is dependent on area and the minor axis as well as the major axis. The judgment matrix are a is shown in Table 3.

In Table 3, feature is wheat seed features, *A* is area, *P* is perimeter, *C* is circularity, *E* is elongation, and *R* is rectangularity.

Because we do not know the extent of the influence of each characteristic parameter on wheat seed grading, the coefficients of the judgment matrix are for test and will be adjusted according to classification results.

From Table 3, paired comparison matrix is as follows:

(6) $$\begin{array}{c} A=\left[\begin{array}{ccccc} 1 & 1 & 3 & 1 & 3\\ 1 & 1 & 5 & 1 & 1\\ \frac{1}{3} & \frac{1}{5} & 1 & 1 & 1\\ 1 & 1 & 1 & 1 & 1\\ \frac{1}{3} & 1 & 1 & 1 & 1 \end{array}\right]. \end{array}$$

Attention: *A* should satisfy the following properties: *a*_*ij*_ > 0, *a*_*ij*_ = 1/*a*_*ji*_, and *a*_*ii*_ = 1; *A* is called positive reciprocal matrix.

#### 4.3.3. Calculating the Sum of Weight by Single Criterion

*Step 1* (elements of *A* are normalized by column). Consider

(7) $$\begin{array}{c} \overset{n}{\underset{i=1}{\sum }}a_{i1}=\frac{11}{3},\;\;\overset{n}{\underset{i=1}{\sum }}a_{i2}=\frac{21}{5},\;\;\overset{n}{\underset{i=1}{\sum }}a_{i3}=11,\\ \overset{n}{\underset{i=1}{\sum }}a_{i4}=5,\;\;\overset{n}{\underset{i=1}{\sum }}a_{i5}=7. \end{array}$$

Every column element is divided by the summation of relative column, and the result is as follows:

(8) $$\begin{array}{c} \left[\begin{array}{ccccc} \frac{3}{11} & \frac{5}{21} & \frac{3}{11} & \frac{1}{5} & \frac{3}{7}\\ \frac{3}{11} & \frac{5}{21} & \frac{5}{11} & \frac{1}{5} & \frac{1}{7}\\ \frac{1}{11} & \frac{1}{21} & \frac{1}{11} & \frac{1}{5} & \frac{1}{7}\\ \frac{3}{11} & \frac{5}{21} & \frac{1}{11} & \frac{1}{5} & \frac{1}{7}\\ \frac{1}{11} & \frac{5}{21} & \frac{1}{11} & \frac{1}{5} & \frac{1}{7} \end{array}\right]. \end{array}$$

*Step 2.* Add the normalized rows together and then the vector is divided by *n*. *n* is the matrix order, and in current model *n* is 5. Weight vector *ω* is as follows:

(9) $$\begin{array}{c} \omega =\left[\begin{array}{c} 0.2824\\ 0.2616\\ 0.1144\\ 0.1889\\ 0.1525 \end{array}\right]. \end{array}$$

*Step 3* (test the consistency of judgment matrix). Consistency test is designed to test the degree of importance of coordination between various elements. Only to avoid critical contradictions (e.g., *A* is more important than *B*; *B* is more important than *C*; and *C* is more important than *A* also), the judgment matrix can be normalized; otherwise the matrix must be rebuilt.

#### 4.3.4. Comprehensive Calculation of Wheat Seeds Feature Weights

*Step 1* (normalize the feature weights). We view the feature vector of wheat seed judgment matrix as feature weights *ω*. It determines the summation of the productions that *ω* is multiplied, respectively, by perimeter, area, elongation, circularity, and rectangularity of wheat seed as total weight value. To make degree of influence about the same among area *A*, perimeter *P*, elongation *E*, circularity *C*, and rectangularity *R*, all above 5 coefficients should be normalized to ensure that the final evaluation is a comprehensive evaluation. Consider *A*′ = *A*/*A*_0_, *P*′ = *P*/*P*_0_, *E*′ = *E*/*E*_0_, *C*′ = *C*/*C*_0_, and *R*′ = *R*/*R*_0_. Among them, *A*_0_, *P*_0_, *E*_0_, *C*_0_, and *R*_0_ are the max value of sample feature. The normalized data are shown in Table 4.

*Step 2* (comprehensive calculation of wheat seeds feature). Comprehensive evaluation is the summation of the productions that *ω* is multiplied, respectively, by perimeter, area, elongation, circularity, and rectangularity of wheat seed. Comprehensive evaluation is marked as *S*

(10) $$\begin{array}{c} S=\omega *\left(A_{0}+P_{0}+E_{0}+C_{0}+R_{0}\right). \end{array}$$

A comprehensive evaluation coefficient of the samples is shown in Table 5.

*Step 3*. After sorting, comprehensive evaluation coefficient is shown in Table 6.

### 4.4. Experiment Result

Compared with actual situation, the numbers of excellent seeds in Figure 1 are 4, 8, 9, 12, 13, and 14; the numbers of good seeds are 1, 6, 10, and 11; the numbers of badness are 3, 2, 5, and 7. It adopts 0.8 or more as excellent classification line and 0.7 or less as bad classification line. Compared with the experimental results, there is error number 1 seed, the overall error rate of 7.1%. By the same way, the error rate is less than 5% for different number of wheat and different forms of placement.

## 5. Conclusions

Wheat seed grading with digital image processing provide further possibilities for the agricultural automation. It helps the manual classification of wheat seed, reduces the amount of labor, and improves the quality of wheat seed. The paper discusses the feature value of wheat seeds and calculates the comprehensive evaluation by AHP. According to the sorting result of comprehensive evaluation, wheat seed can be graded. Compared with the fact, the overall accuracy rate of wheat seed is more than 95%, which meets the actual needs. The method can be extended to the other granular agricultural classification test.

## Figures and Tables

**Table 1:** Characteristic values of wheat seeds.

Num	*A*	*P*	*C*	*E*	*R*
1	475830	58	1.7775	1	157.2992
2	408765	71	1.019	1.5385	174.6859
3	391425	67	1.0957	2.3103	201.4539
4	422280	76	1.2182	1.102	159.5918
5	581400	72	1.2649	1.4783	185.8696
6	472770	67	1.3235	1.2766	167.6489
7	229755	67	0.6432	1.1463	119.2294
8	557940	75	1.2464	1.8421	209.7519
9	640815	78	1.3236	1.1017	167.0965
10	577320	67	1.6161	1.0714	171.8214
11	451350	73	1.0643	1.9412	201.1364
12	550035	89	0.8726	2.1714	206.7801
13	555135	83	1.0126	1.875	185.045
14	563550	69	1.4875	1.4	198.7831

**Table 2:** Importance value.

Importance value	Meaning
1	Compared to the two elements, they are equally important
3	Compared to the two elements, the former is slightly more important than the latter
4	Compared to the two elements, the former is obviously more important than the latter
7	Compared to the two elements, the former is strongly more important than the latter
9	Compared to the two elements, the former is extremely more important than the latter
2, 4, 6, 8	They represent the middle values of the above judgments
Reciprocal	If the importance ration of *I* and *J* is *a*_*ij*_, the importance ration of *J* and *I* is *a*_*ji*_ = 1/*a*_*ij*_

**Table 3:** The weight ratio between characteristic values.

Feature	*A*	*P*	*C*	*E*	*R*
*A*	1	1	3	1	3
*P*	1	1	5	1	1
*C*	1/3	1/5	1	1	1
*E*	1	1	1	1	1
*R*	1/3	1	1	1	1

**Table 4:** Features normalized.

Num	*A*	*P*	*C*	*E*	*R*
1	0.7425	0.6517	1	0.4328	0.7499
2	0.6379	0.7978	0.5733	0.6659	0.8328
3	0.6108	0.7528	0.6164	1	0.9604
4	0.659	0.7416	0.6853	0.477	0.7609
5	0.9073	0.8539	0.7116	0.6399	0.8861
6	0.7378	0.7528	0.7446	0.5526	0.7993
7	0.3585	0.7528	0.3619	0.4962	0.5684
8	0.8707	0.8427	0.7012	0.7973	1
9	1	0.8764	0.7446	0.4769	0.7966
10	0.9009	0.7528	0.9092	0.4637	0.8192
11	0.7043	0.8202	0.5988	0.8402	0.9589
12	0.8583	1	0.4909	0.9399	0.9858
13	0.8663	0.9326	0.5697	0.8116	0.8822
14	0.8794	0.7753	0.8368	0.606	0.9477

**Table 5:** Comprehensive evaluation coefficient.

Number	Coefficient
1	0.6907
2	0.7072
3	0.7753
4	0.817
5	0.6646
6	0.7167
7	0.52
8	0.8497
9	0.8084
10	0.7679
11	0.7869
12	0.888
13	0.8416
14	0.8059

**Table 6:** Wheat seeds sorting evaluation coefficient.

Number	Coefficient	Rank
12	0.888	1
8	0.8497	2
13	0.8416	3
4	0.817	4
9	0.8084	5
14	0.8059	6
11	0.7869	7
3	0.7753	8
10	0.7679	9
6	0.7167	10
2	0.7072	11
1	0.6907	12
5	0.6646	13
7	0.52	14
